# SEXUAL ORIENTATION AS MULTIDIMENSIONAL: AGE AND GENDER DIFFERENCES IN SEXUAL MINORITY OLDER ADULTS

**DOI:** 10.1093/geroni/igz038.1114

**Published:** 2019-11-08

**Authors:** Eve Z Root, Grace Caskie, Bethany Detwiler, Nicole L Johnson

**Affiliations:** Lehigh University, Bethlehem, Pennsylvania, United States

## Abstract

Recommendations to conceptualize sexual orientation as a continuum and as multidimensional rather than one dichotomous variable (e.g., DeBlaere et al., 2010; Kinsey et al., 1948) have been largely unexplored in sexual minority older adults, including how these dimensions might differ by age and gender. In this study, participants indicated their sexuality using three continua representing (1) attraction in general, (2) emotional attraction, and (3) physical attraction. Possible responses ranged from 0=exclusively opposite sex to 7=exclusively same sex. The current sample included 187 participants (50-86 years; 73 men, 114 women) self-identifying their sexual attraction in general as not exclusively to the opposite sex. Age groups were 50-55 (n=56), 56-64 (n=84), and 65-86 (n=47) years. MANOVA results indicated a significant multivariate age group by gender interaction (p=.040) that was significant for all three attraction variables---attraction in general (p=.035), emotional attraction (p=.010), and physical attraction (p=.029). In the 50-55 age group, the average response for physical attraction was closer to exclusively same sex for men than for women. For the 56-64 age group, the average response for attraction in general and emotional attraction was closer to exclusively same sex for women than men. Among those 65+, women responded closer to exclusively same sex than men only for emotional attraction. Gender differences on all three sexual attraction continua were not consistent across age groups, which may reflect a more fluid and complex understanding of sexuality in older LGB adults. Future studies should consider using multidimensional and continuous variables when measuring sexual orientation.

